# Constructing a semantic predication gold standard from the biomedical literature

**DOI:** 10.1186/1471-2105-12-486

**Published:** 2011-12-20

**Authors:** Halil Kilicoglu, Graciela Rosemblat, Marcelo Fiszman, Thomas C Rindflesch

**Affiliations:** 1 Lister Hill National Center for Biomedical Communications, National Library of Medicine, Bethesda, MD, USA; 2 Department of Computer Science and Software Engineering, Concordia University, Montreal, QC, Canada

## Abstract

**Background:**

Semantic relations increasingly underpin biomedical text mining and knowledge discovery applications. The success of such practical applications crucially depends on the quality of extracted relations, which can be assessed against a gold standard reference. Most such references in biomedical text mining focus on narrow subdomains and adopt different semantic representations, rendering them difficult to use for benchmarking independently developed relation extraction systems. In this article, we present a multi-phase gold standard annotation study, in which we annotated 500 sentences randomly selected from MEDLINE abstracts on a wide range of biomedical topics with 1371 semantic predications. The UMLS Metathesaurus served as the main source for conceptual information and the UMLS Semantic Network for relational information. We measured interannotator agreement and analyzed the annotations closely to identify some of the challenges in annotating biomedical text with relations based on an ontology or a terminology.

**Results:**

We obtain fair to moderate interannotator agreement in the practice phase (0.378-0.475). With improved guidelines and additional semantic equivalence criteria, the agreement increases by 12% (0.415 to 0.536) in the main annotation phase. In addition, we find that agreement increases to 0.688 when the agreement calculation is limited to those predications that are based only on the explicitly provided UMLS concepts and relations.

**Conclusions:**

While interannotator agreement in the practice phase confirms that conceptual annotation is a challenging task, the increasing agreement in the main annotation phase points out that an acceptable level of agreement can be achieved in multiple iterations, by setting stricter guidelines and establishing semantic equivalence criteria. Mapping text to ontological concepts emerges as the main challenge in conceptual annotation. Annotating predications involving biomolecular entities and processes is particularly challenging. While the resulting gold standard is mainly intended to serve as a test collection for our semantic interpreter, we believe that the lessons learned are applicable generally.

## Background

Large-scale information extraction (IE) from scientific literature is increasingly used to support advanced knowledge management and discovery systems [1-3]. The utility of such systems depends on the quality of the extracted information. Manually annotated gold-standard corpora are critical for evaluating the accuracy and usefulness of information extraction systems [4]. In the biomedical domain, various corpora annotated for semantic phenomena have been constructed in recent years; annotations range from named entities [4-7], to semantic relations, such as protein-protein interactions [8,9], protein/gene/RNA relationships [10], disease-treatment relations [11], clinical relations [12], biological events [13], and gene regulation events [14]. More recently, the notion of "silver standard" has also been introduced [15], referring to harmonization of automated system annotations, as a proxy to labor-intensive gold standard annotation.

The gold standard corpora have often focused on text drawn from a narrow subdomain, adopting a particular semantic representation, addressing a small set of semantic types and aiming to provide training and evaluation support for specialized IE systems. These corpora differ with respect to their level of granularity and whether there is an ontological basis to the entity and relationship types used. For example, one of the most popular corpora in recent years has been the GENIA event corpus [13], drawn from the scientific literature on transcription factors in human blood cells. It is based on the notion of biological *events*, uses a few dozen Gene Ontology (GO) [16] event types and has been the basis for recent biological event extraction systems as well as two BioNLP Shared Task competitions [17,18]. The generally narrow focus of such corpora and their specific representation formalisms render them largely unsuitable for evaluating IE systems using different formalisms or resources.

We have been developing a semantic interpreter, SemRep [19], which extracts content from biomedical text in the form of semantic predications. A semantic predication is a logical subject-predicate-logical object triple whose elements are drawn from the UMLS knowledge sources [20]; the subject and object pair corresponds to UMLS Metathesaurus concepts and the predicate to a relation type in an extended version of UMLS Semantic Network. While the UMLS Semantic Network has not been designed as an ontology in a strict sense, the extended version that SemRep uses [21] serves as an ontological resource: it defines a domain model consisting of concept types (*semantic types*), relation types (*ontological predicates*) and the relationships that can hold between concept types (*ontological predications*). Each semantic predication extracted by SemRep is an *instantiation *of an ontological predication. We refer to this extended version of the UMLS Semantic Network as the *SemRep ontology *henceforth.

SemRep extracts a range of predicates relating to clinical medicine (e.g. TREATS, DIAGNOSES, ADMINISTERED_TO, PROCESS_OF), substance interactions (e.g., INTERACTS_WITH, INHIBITS, STIMULATES), genetic etiology of disease (e.g., ASSOCIATED_WITH, CAUSES, PREDISPOSES), and pharmacogenomics (e.g., AFFECTS, AUGMENTS, DISRUPTS). For example, the program identifies the semantic predications in (2) from the input text in (1). Arguments of the semantic predications (subject and object) have the form *ConceptIdentifier: ConceptName (ConceptSemanticType)*. Textual mentions corresponding to the arguments are in bold, and those corresponding to the predicates are underlined in (1).

*(1) **MRI **revealed a **lacunar infarction **in the left **internal capsule***.

(2) C0024485: Magnetic Resonance Imaging (Diagnostic Procedure)-DIAGNOSES-C0333559: Infarction, Lacunar (Disease or Syndrome)

C0152341: Internal Capsule (Body Part, Organ, or Organ Component)-LOCATION_OF-C0333559: Infarction, Lacunar (Disease or Syndrome)

SemRep processing is supported by an underspecified syntactic analysis based on the UMLS SPECIALIST Lexicon [22] and the MedPost part-of-speech tagger [23]. MetaMap [24] is used to map simple noun phrases to UMLS Metathesaurus concepts. Entrez Gene [25] serves as a supplementary source to the UMLS Metathesaurus with respect to gene/protein terms, which are identified using ABGene [26], in addition to MetaMap. Indicator rules map syntactic phenomena, such as verbs, nominalizations, prepositions, and modifier-head structure in the simple noun phrase, to ontological predicates from the SemRep ontology. SemRep currently uses the 2006AA release of the UMLS knowledge sources, due to the prevalence of ambiguity in later releases.

The lack of a suitable, manually annotated gold standard corpus has so far precluded a formal evaluation of SemRep (for focused, task-based evaluations, see [27-29]); system improvements and modifications have been informally evaluated through error analysis. A formal evaluation requires conceptual annotation with respect to the UMLS; that is, text fragments need to be mapped to concepts and relations in the UMLS, which provides a formal representation of domain knowledge. Considering that the UMLS Metathesaurus, the basis for conceptual information, consists of 92 source vocabularies and more than 1.2 million concepts (in 2006AA release), it is clear that such conceptual annotation is an extremely challenging task.

Large-scale conceptual annotation is not generally attempted in the biomedical domain. In fact, apart from the recent CRAFT corpus [4] and the CLEF corpus [12], we are not aware of any such annotation work. The ontology-based semantic annotation of the CRAFT Corpus [4] concentrates on biomolecular entities and processes, including gene/gene products, chemicals, sequence types, molecular functions, and cellular components. Ninety-seven full-text articles were annotated with concepts from eight terminologies, six of them from the OBO library [30]. They report that relationship annotation between concepts is ongoing work. Intended for clinical IE research, the CLEF corpus [12] annotates information about clinical entities and the relations between them, along with temporal information. It is limited to clinical notes and reports of deceased patients who had a diagnosis of neoplasms. CLEF builds on a relevant subset of UMLS concepts and relations as domain knowledge. Some semantic types are conflated and relations (predicates) renamed in accordance with the goals of the project. For instance, CLEF semantic type Condition includes symptoms, complications, functions, diagnosis, and problems, conflating several UMLS semantic types. In contrast to these manual conceptual annotation efforts, Jimeno et al. [7] semi-automatically created a small corpus annotated with disease concepts from UMLS Metathesaurus. Their semi-automatic methodology involves domain expert assessment of disease concepts identified by MetaMap, a dictionary lookup method, and a statistical method.

In this article, we present an annotation study in which we annotated 500 sentences from MEDLINE abstracts with 1371 semantic predications. Our annotation follows the conceptual annotation paradigm and adopts the entire UMLS as the domain model. Furthermore, we do not limit ourselves to text from a specific subdomain or adopt specific terminologies, in contrast to more focused efforts such as CLEF and CRAFT. While our methodology bears some similarities to the CLEF methodology in terms of the domain model and the guidelines, we use fine-grained UMLS semantic types, rather than coarse semantic groups, allowing for more flexibility. With respect to our ongoing research, the resulting gold standard reference is mainly intended to (a) facilitate comparison between SemRep releases and (b) guide further system development by allowing annotators to add comments and notes in a standardized manner so that problem areas can be identified. Several limitations of the gold standard reference may include its relatively small size, its sentence-bound annotation, and its binary, UMLS Semantic Network semantic relation formalism, arguably not as rich a semantic representation as predicate-argument structures with semantic roles (PASBio [31], GENIA event corpus [13], GREC [14]). While small corpus size may present challenges for learning purposes, it serves well for our primary goal of evaluation. On the other hand, the semantic predication representation (triples) has been shown to be simple, intuitively accessible, and tractable for large-scale knowledge discovery applications [3]. Furthermore, this representation lends itself readily to the Semantic Web and linked data movements, which aim to encode knowledge in subject-predicate-object triples for large-scale automatic reasoning [32]. Considering the challenges posed by this task, we believe our corpus presents a good first step in a larger-scale conceptual/relational annotation. It can serve as a gold standard reference for evaluating UMLS-based relation extraction systems and the lessons learned can provide guidance for future efforts in this area.

## Methods

We conducted the annotation study in three phases: a) practice annotation phase, b) main annotation phase and c) adjudication phase. Before explaining these phases in more detail, we briefly discuss two fundamental aspects of our study: annotators and interannotator agreement.

### Annotators

Three annotators, all authors of this paper, were involved in the annotation process. The annotators have diverse backgrounds; annotator A is a linguist, B a computer scientist and C a physician/biomedical informatics researcher. All three annotators have natural language processing experience and are experts in the SemRep methodology as well as the UMLS knowledge sources.

Using domain experts as annotators is often considered a good strategy to ensure validity and reliability of annotation. However, the tendency of domain experts to rely on inference due to their background knowledge has also been noted [13]. When annotation is concerned with a relatively narrow biomedical subdomain, it is generally feasible to recruit domain experts as annotators. However, several aspects of our annotation study make it difficult to find such annotators. First, we do not focus on a narrow biomedical subdomain. The consequence is that we need to either recruit experts who are knowledgeable in almost all aspects of biomedicine, or to find tens of annotators who can annotate different sentences on topics of their expertise. Neither option seemed feasible within the scope of our annotation. Furthermore, the two non-physician annotators (A and B) work with biomedical text on a full-time basis, and they expressed comfort with annotation after the practice phase. Secondly, in our annotation study, UMLS expertise is perhaps as crucial as domain expertise, and all three annotators are intimately familiar with the UMLS knowledge sources.

Recruiting domain experts who are also familiar with the UMLS would clearly be even more challenging. We believe that our small team of annotators with their expertise in UMLS and our multi-phase annotation methodology allows us to strike a balance between annotation reliability and validity.

### Interannotator Agreement

The common approach to calculating interannotator agreement on classification tasks is to use *kappa *(*κ*) statistic [33], defined as *κ = (Pr(a) - Pr(e))/(1-Pr(e))*, where *Pr(a) *is the relative observed agreement between annotators, and *Pr(e) *is the chance agreement. Calculating *κ *for semantic predication annotations is non-trivial, since this type of linguistic annotation is not a simple classification task. Semantic predication annotation task can be decomposed into several subtasks for which agreement can be measured independently: (a) finding relation indicators, (b) finding textual mentions of arguments, (c) mapping the relation indicators to ontological predicates, and (d) mapping the textual mentions of arguments to concepts. With the exception of subtask (c), the space of possible annotations is either not clearly defined or very large, making the calculation of *Pr(e) *and therefore *κ*, challenging. For example, consider subtask (a). One way to calculate *κ *for this subtask is to impose constraints on what can be annotated as an indicator. For example, only verbs may be considered as indicators and the number of verbs in a sentence can be used to calculate chance agreement *Pr(e*). We did not impose such constraints since we aimed for breadth of coverage in our annotation study. This leads to an explosion of possible indicator annotations and to the case in which *Pr(e) *essentially approaches zero. In such cases, it has been shown that *κ *approximates F-measure among pairs of annotators [34]. Based on these observations and in line with annotation studies that share similarities with ours [12,14], we adopted F-measure for interannotator agreement. Between two sets of annotations, we calculated it as the F-score of one set of annotations, when the second is taken as the gold standard.

### Practice Phase

**Figure 1:**
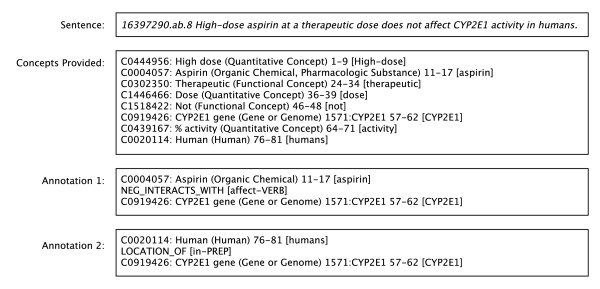
**Sample annotation provided to the annotators**. The sample annotation provided to the annotators before the practice phase. The first line of the annotation corresponds to the subject, the second line to the predicate and the third line to the object of the predication. Some fields are not shown for readability.

For the practice annotation phase, we randomly selected 50 sentences (average of 29.8 tokens per sentence) from 50 MEDLINE abstracts published in the last 10 years. These sentences were extracted from the database of Semantic MEDLINE [27], a Web application to manage the results of PubMed searches, and were manually checked by the first author to ensure correct sentence segmentation. All three annotators participated in the practice annotation phase, each annotating the same set of 50 sentences. Given the annotators' familiarity with SemRep, minimal guidance for annotation was provided at this phase and the annotators were asked to annotate semantic predications expressed in the sentences, the textual mentions of their arguments and the predicate and the indicator type (i.e., whether the predicate is a verb (VERB), preposition (PREP), nominalization (NOM), participle (PART), etc.). To lighten the burden of finding an appropriate UMLS Metathesaurus concept corresponding to a textual mention, UMLS Metathesaurus concepts were extracted from these sentences using MetaMap [24] and were provided to the annotators (an average of 9.86 concepts per sentence). The guidance at this phase consisted of the following items:

1. A list of core ontological predicates relevant to SemRep and their definitions from the UMLS. For ontological predicates that are not part of the official UMLS Semantic Network but are in the SemRep ontology, we used our own definitions. We provide these definitions in the Appendix.

2. An inventory of predicate types (preposition, nominalization, verb, etc.), also provided in the Appendix.

3. A sample sentence annotation, provided in the Appendix and illustrated in Figure 1.

4. Basic instructions consisted of the following:

a) Annotation should be restricted to semantic predications involving the core ontological predicates that are provided. Other predicates, even though they may be legitimate, should be ignored. On the other hand, the annotators are not restricted to follow the ontological predications that exist in the SemRep ontology.

b) The UMLS concepts extracted by MetaMap are not necessarily the best possible mappings. In addition, MetaMap may be unable to find a mapping. When in doubt, the annotator should try to find a concept that better matches the text (a UMLS Metathesaurus concept or an Entrez Gene term) using the UMLS Terminology Services (UTS [35]) or Entrez Gene [36], keeping in mind that SemRep currently uses the 2006AA version of the UMLS knowledge sources.

c) The annotation should be text-bound. That is to say, domain knowledge and inference should play a minimal role in annotation and the annotator should be concerned with what is explicitly stated in the text. To ensure this, the annotator should explicitly indicate the textual mentions that provide the basis for the annotation (those indicating the subject, object and the predicate), as well as the indicator type for the annotation.

Providing only minimal guidance, we aimed to identify the major challenges in annotating predications and find ways to deal with them in the main annotation phase. This first phase was collaborative: the annotators were free to discuss sentences, concerns and difficulties and develop solutions.

We chose not to use a particular annotation tool, since none of the existing tools fully met our needs, especially with respect to access to terminological resources. We decided not to develop a study-specific, in-house annotation tool due to time constraints. Instead, the annotators were instructed to simply type semantic predications in a text document, along with the textual mentions that trigger the predication components. Then, based on the results of the first phase, annotators were provided scripts that recognized formatting, spelling errors, and inconsistencies in annotation. These scripts were used by annotators in subsequent phases and helped them resolve such errors.

The practice annotation phase was completed in three weeks. After this phase concluded, the first author analyzed each annotation set to identify annotation patterns. The analysis results were then discussed among the annotators and served to refine the guidelines for the second phase. We present these refinements in the Results section. We also computed a baseline interannotator agreement between pairs of annotators. To assess agreement with respect to textual mentions vs. conceptual information, we calculated interannotator agreement using two criteria:

*a. strict equivalence *criterion, where for two semantic predications to be considered equal, their subject-predicate-object triples must match exactly.

*b. relaxed equivalence *criterion, where the exact match of the textual mentions of the arguments and of the predicate that establishes their relationship are considered sufficient for equality. In other words, conceptual match is not required between predication elements.

### Main Annotation Phase

In the main annotation phase, two annotators (A and B) annotated a set of 500 randomly selected sentences drawn from 308 MEDLINE abstracts from the past 10 years (average of 27.9 tokens per sentence). Similar to the practice phase, sentences were drawn from the Semantic MEDLINE database, were checked for integrity, and UMLS Metathesaurus concepts extracted by MetaMap were provided for reference (9.09 concepts per sentence). The annotators worked independently in this phase, which concluded in eight weeks.

Based on our observations from the practice phase, we extended the equivalence criteria underlying interannotator agreement in two ways:

a. *Predication equivalence (PE): *A pair of distinct semantic predications may be considered equivalent under specific conditions when one inverts the arguments of the other and the predicates correspond to certain types. For instance, a predication X-LOCATION_OF-Y may be considered equivalent to Y-PART_OF-X predicates when arguments (X or Y) correspond to biomolecular entities.

b. *Gene/gene product correspondence (GP): *A pair of concepts may be considered equivalent when one corresponds to a gene term and the other corresponds to its gene product. For instance, the concept "C0287531: DUSP1 protein, human" (Amino Acid, Peptide, or Protein, Enzyme) is considered equivalent to "C1333257: DUSP1 gene" (Gene or Genome).

We also assessed the effect on annotation of domain knowledge provided to the annotators. For this purpose, we distinguished between two types of domain knowledge, *conceptual *and *relational*, and measured interannotator agreement on a subset of predications based on the following availability criteria, illustrated in (3-5) below:

a. *Availability of conceptual knowledge (CK): *A predication fulfills this criterion if both the subject and object arguments were extracted by MetaMap and, thus, were provided to the annotators.

b. *Availability of relational knowledge (RK): *A predication fulfills this criterion if it is sanctioned by the SemRep ontology. In other words, it corresponds to an existing ontological predication.

c. *Availability of conceptual and relational knowledge (CRK): *A predication fulfills this criterion if it satisfies both (a) and (b), the previous two criteria.

For illustration, consider the sentence fragment in (3). Relevant concepts identified by MetaMap are given in (4), and an annotated predication in (5).

(3) ... *UDP-Glc is required in the synthesis of proteoglycans*. ...

(4) C0041986: Uridine Diphosphate (Biologically Active Substance; Carbohydrate; Nucleic Acid, Nucleoside, or Nucleotide)

C0008556: Chromatography, Gas-Liquid (Laboratory Procedure)

C0033692: Proteoglycan (Amino Acid, Peptide, or Protein; Biologically Active Substance)

(5) C0041988: Uridine Diphosphate Glucose (Biologically Active Substance)-PRODUCES-C0033692: Proteoglycan (Amino Acid, Peptide, or Protein)

While the predication in (5) is correct, it does not fulfill criterion (a) above because MetaMap fails to identify the subject "C0041988: Uridine Diphosphate Glucose (Biologically Active Substance)," an argument absent in (4). It also fails to fulfill criterion (b) because the corresponding ontological predication "Biologically Active Substance-PRODUCES-Amino Acid, Peptide, or Protein" is not licensed by the current SemRep ontology. Since neither criterion (a) nor (b) are met, it follows that the predication does not fulfil criterion (c), either. As a result, the predication in (5) is not included when interannotator agreement calculation is restricted by any of the available domain knowledge criteria above.

### Adjudication

Finally, the third annotator (C) examined the annotations provided by each annotator and adjudicated them to create the current gold standard. During this phase, annotator C was free to discuss the annotations with the actual annotator to understand his/her reasoning. This phase concluded in four weeks.

## Results

In this section, we provide detailed information regarding different phases of the annotation, including the number of semantic predications annotated, their distribution by ontological predicate types, and indicator types. We report strict and relaxed interannotator agreement using various equivalence criteria and domain knowledge perspectives. For the main annotation phase, we measure interannotator agreement at the ontological predicate and ontological predication level, highlighting some of the annotation difficulties with respect to biomedical subdomains. Examining annotation differences in the practice phase, we refined our guidelines for the main annotation phase and extended interannotator agreement measures, which we also discuss in this section.

### Practice Phase

In this first phase, 50 sentences were annotated, with an average of 2.68 semantic predications per sentence. We show the number of predications annotated by each annotator at this phase in Table 1, and the most frequently annotated ontological predicates with their annotation frequency in Table 2. Indicator types that signal predications are provided in Table 3, which shows that verbal predicates are considered at similar rates among annotators, while there are larger gaps in consideration of other types.

**Table 1:** Overall semantic predication statistics in the practice phase on the set of 50 sentences annotated by all three annotators

Annotator	# of Predications	Per sentence	Max. per sentence
A	130	2.60	12

B	156	3.12	9

C	116	2.32	11

**Table 2:** Top ontological predicates and their annotation frequency in the practice phase

Predicate	Average Count	%	A	B	C
LOCATION_OF	19.3	14.4	26	19	13

PROCESS_OF	17.3	12.9	14	28	10

INHIBITS	12.3	9.2	16	7	14

INTERACTS_WITH	11.3	8.4	13	4	17

ISA	10.3	7.7	12	9	10

PART_OF	7.3	5.4	3	14	5

TREATS	7	5.2	5	11	5

CAUSES	7	5.2	7	5	9

**Table 3:** Top indicator types and their annotation frequency in the practice phase

Predicate Type	Average Count	A	B	C
PREP	37	39	47	25

VERB	27	26	25	30

NOM	24	19	23	30

MOD_HEAD	19.3	14	32	12

PART	12.7	21	10	7

Interannotator agreement was fair to moderate in this phase, as shown in Table 4. The highest agreement occurred between annotators A and C (0.475), consistent with the largely similar patterns they exhibited in the types of ontological predicates they annotated, as shown in Table 2. Unsurprisingly, the interannotator agreement increases, albeit at different rates, when equivalence of textual mentions is also considered as the basis for agreement (*relaxed equivalence*) as well as the conceptual equivalence (*strict equivalence*).

**Table 4:** Inter-annotator agreement (IAA) in the practice phase, calculated as F-measure among pairs of annotators

Pair	A-B	A-C	B-C
IAA (*strict*)	0.415	0.475	0.378

IAA (*relaxed*)	0.428	0.500	0.434

### Refining the Guidelines

The results of the practice phase helped us identify several aspects of annotation that are challenging. These were discussed among the annotators and several additions and clarifications were made in the annotation guidelines. These included the following:

#### Selecting appropriate UMLS Metathesaurus concepts

As stated earlier, we provided the annotators UMLS Metathesaurus concepts identified by MetaMap for reference. After the first phase, our analysis revealed that annotation behavior was diverse with regard to these MetaMap-provided concepts. One annotator almost exclusively relied on them, while the other two more extensively utilized the UMLS to find more adequate concepts. In addition, one of the annotators considered Metathesaurus concepts from newer UMLS releases. In the light of discussions over these differences, annotators were instructed to use the following decision criteria in selecting an appropriate concept:

1. If the concept identified by MetaMap adequately describes the textual mention, use it.

2. A concept that is clearly more general than the one stated in the text (that is, a hypernymic (ISA) relation holds between them) cannot be used as a replacement.

3. If MetaMap does not associate any concept with the textual mention OR if the associated concept seems inadequate, try to find a UMLS 2006AA concept that is appropriate.

4. When there is no corresponding UMLS 2006AA concept, a concept from a newer UMLS release may be used, provided that it does not violate the decision criterion (2) above.

#### Finding Entrez Gene terms

SemRep uses Entrez Gene [25] as a supplementary vocabulary to UMLS Metathesaurus when it identifies gene/protein terms. The mapping to Entrez Gene terms is achieved via pattern matching in SemRep. When available, these symbols were also provided to annotators in the practice phase. Similar to their reactions to MetaMap-supplied UMLS concepts, annotators also relied on Entrez Gene terms to varying degrees. One annotator tried to find Entrez Gene terms for all gene/protein mentions, whether or not a corresponding UMLS concept was found, and disambiguate model organisms using the context surrounding the textual mention, while another completely ignored Entrez Gene terms. We identified two issues regarding these terms: (a) when is it required to annotate a textual mention with an Entrez Gene term? (b) is disambiguation with respect to model organisms necessary? We resolved these issues by concluding that the UMLS is the primary knowledge source for SemRep, and a gene/protein mention needs to be annotated with an Entrez Gene term only if no corresponding UMLS concept is found. Further, we decided that Entrez Gene terms should be limited to those in the *Homo sapiens *taxon, since SemRep currently only considers this taxon, and context beyond individual sentences may need to be taken into account for determining the model organism.

#### Semantic Network Ontological Predicates

The practice phase revealed some confusion with respect to the difference between an ontological predicate and its textual mention. For instance, for the fragment "... an association of 5-HTTLPR with intensity dependence of auditory-evoked potentials...", two annotators annotated the semantic predication "C0170657: serotonin transporter (Biologically Active Substance)-ASSOCIATED_WITH-C0015215:Auditory Evoked Potentials (Organism or Tissue Function)." The ontological predicate ASSOCIATED_WITH is used in SemRep in a restricted sense, referring to only gene-disease association. Although the semantic type of the object in the predication is not a disorder, the annotators used this ontological predicate because they were influenced by the choice of textual mention "association." The difference was made explicit in the guidelines.

We further clarified the definitions of several ontological predicates, taking into account how they are conceptualized in the SemRep ontology. For instance, we noted that comparative predicates (COMPARED_WITH, HIGHER_THAN, LOWER_THAN, SAME_AS) are limited to substance and therapeutic procedure semantic types for the time being, while PROCESS_OF is limited to disorder subjects. We also distinguished INTERACTS_WITH/AFFECTS and INHIBITS/DISRUPTS predicate pairs more explicitly: INTERACTS_WITH and INHIBITS relations hold between substances, while AFFECTS and DISRUPTS take processes as objects.

#### Hypernymic Relations

There was disagreement over what constitutes a hypernymic (ISA) relation. Problematic annotations included "C0050940: Lansoprazole (Organic Chemical)-ISA-C0599473: Enantiomer (Chemical Viewed Structurally)" for the noun phrase "lansoprazole enantiomers" and "C1443775: Epidermal growth factor receptor inhibitor (Pharmacologic Substance)-ISA-C0450442:Agent (Chemical Viewed Functionally)" for the fragment "Two oral EGFR inhibitors ... are small-molecule agents ...." We concluded our discussion on this topic by distinguishing between taxonomic relations that pertain to structural aspects (as in the former predication above) and other kinds of taxonomic relations, including those pertaining to functional aspects (the latter). It was clarified in the guidelines that structural taxonomic relations are not hypernymic.

### Extending Interannotator Agreement Calculation

As briefly mentioned earlier, based on analysis of the agreement results in the practice phase, we extended equivalence criteria for interannotator agreement calculation. We describe these extensions in more detail in this section.

#### Equivalence of Semantic Predications (PE)

We found that two distinct semantic predications derived from the same textual mentions may be too close in meaning to select one over the other. For instance, we identified cases where one annotator used a predication with the predicate LOCATION_OF, while the other preferred one with the predicate PART_OF and inverse arguments. For the textual fragment "... alleles in two cell lines", one annotator annotated "C0002085: Alleles (Gene or Genome)-PART_OF-C0007600: Cell Line (Cell)", while the other annotated "C0007600: Cell Line (Cell)-LOCATION_OF-C0002085: Alleles (Gene or Genome)." Such similarity in meaning is partly rooted in the UMLS Semantic Network. For instance, the ontological predication "Virus-LOCATION_OF-Biologically Active Substance" is a valid ontological predication, as is one with inversion of these arguments and PART_OF as predicate ("Biologically Active Substance-PART_OF-Virus"). In addition, some indicator expressions can be ambiguous with respect to their meaning; for instance, the preposition "in" may map to either predicate in this example. Examining instances of the LOCATION_OF/PART_OF variation in the practice phase, we concluded that at the biomolecular level, the difference in meaning is blurred to the extent that two semantic predications may be considered equivalent for interannotator agreement calculation. However, we also noted that there are clear exceptions. For example, consider the sentence fragment "... truncated Bid (tBid), which translocates to mitochondria ...". While the predication "C0026237: Mitochondria (Cell Component)-LOCATION_OF-C1144558: tBid Protein (Amino Acid, Peptide, or Protein)" seems acceptable for this fragment, its counterpart with the predicate PART_OF ("C1144558: tBid Protein (Amino Acid, Peptide, or Protein)-PART_OF-C0026237: Mitochondria (Cell Component)") does not. To handle such exceptions, we manually judged the cases in which there was agreement due to this equivalence criterion for correctness as a post-processing step to interannotator agreement calculation and corrected the agreement score accordingly.

A situation similar to LOCATION_OF/PART_OF equivalence concerns PRODUCES/PART_OF predicates and the verbal indicator "express" and its derivations. A relation indicating gene expression events does not currently exist in the SemRep ontology, leading one annotator to use PART_OF consistently for such events, and the other to use PRODUCES. In the fragment "The expressions of c-Myc, Ki-67, MMp-2 ... in cancer tissues", one annotator chose "C1334508: MKI67 gene (Gene or Genome)-PART_OF-C0040300: Body tissue (Tissue)" and the other "C0040300: Body tissue (Tissue)-PRODUCES-C1334508: MKI67 gene (Gene or Genome)." We treat these cases as equivalent as well. Interannotator agreement using these two equivalence criteria is denoted as PE below.

#### Correspondence of Gene and Gene Products (GP)

In the molecular biology literature, gene names are often used to denote gene products (proteins), leading to term ambiguity. For instance, in the fragment "TNFR1/Fas engagement results in the cleavage of cytosolic Bid to ...", without knowing the context, it is difficult to determine whether TNFR1, Fas or Bid refer to genes or gene products. This ambiguity extends to the UMLS Metathesaurus as well. For instance, Bid maps to "C1332410: BID gene (Gene or Genome)" and "C0531588: BID protein (Amino Acid, Peptide, or Protein, Biologically Active Substance)." On the other hand, TNFR1 is mapped to "C0255808: tumor necrosis factor receptor 1A (Amino Acid, Peptide, or Protein, Receptor)" as well as "C1363984: TNFRSF1A gene (Gene or Genome". We extended our interannotator agreement calculation to take this into account and considered it a match when one predication involves the concept name "X gene", while the other involves the name "X protein*", where * indicates wildcard characters. This correspondence criterion accommodates the former (the simple case of Bid), while the equivalence in the case of TNFR1 is ignored. Although the two concepts ("tumor necrosis factor receptor 1A" vs. "TNFRSF1A gene") corresponding to the textual mention ("TNFR1") are considered Related Terms in UMLS, it is difficult to establish their correspondence from their concept names alone. Correspondence of this type is denoted as GP below.

#### Limiting Agreement Calculation by Available Domain Knowledge (CK/RK/CRK)

To assess the difficulty of annotation due to the open-ended, exploratory nature of our annotation, we also limited interannotator agreement calculation based on the availability of conceptual knowledge to the annotator (CK) as well as that of relational knowledge (RK) and both (CRK), as mentioned earlier. Our intuition in limiting calculation to a subset of annotated predications was that there would be more substantial agreement when the annotator chooses concepts and ontological predications from a predefined set.

### Main Annotation Phase

Two annotators (A and B) were involved in the main annotation phase. The average number of semantic predication annotations per sentence was slightly lower than that in the practice phase (2.64 vs. 2.68, respectively); one annotator (A) was relatively consistent between the practice and main annotation phases, while the other (B) annotated significantly fewer in the main phase, as shown in Table 5.

**Table 5:** Overall semantic predication statistics in the main annotation phase on the set of 500 sentences annotated by two annotators

Annotator	# of Predications	Per sentence	Max. per sentence
A	1293	2.59	24

B	1344	2.69	22

The interannotator agreement results for the main annotation phase are presented in Table 6. With the improved guidelines provided to the annotators, the basic *strict *agreement between the annotators increased from 0.415 to 0.500, while the basic *relaxed *agreement increase was higher: from 0.428 to 0.535. Additionally, we computed interannotator agreement using the extended equivalence criteria (rows 2-4). Adding the predication equivalence criterion (PE) increased the agreement by approximately 3%, while gene/gene product correspondence criterion (GP) provided a small increase of 0.5% overall. Limiting the comparison to conceptual knowledge provided (CK-column 3), the agreement reaches substantial levels with an increase of approximately 12%. Relational domain knowledge alone (RK-column 4) had less effect on agreement (an increase of about 3.5%). With both conceptual and relational knowledge provided to annotators (CRK-column 5), the agreement rises more than 15% in comparison to the basic case. We only show *relaxed *agreement in the basic case, where the increase from the strict counterpart is about 3.5%. We note that this trend was observable in the cases of predication filtering, as well (columns 3-5). We consider *strict *agreement with *base + PE + GP equivalence *as the main agreement criterion in the rest of the paper (0.536).

**Table 6:** Interannotator agreement (A-B) in the main annotation phase, calculated as F-measure among the pair of annotators

Equivalence Criteria	*strict (relaxed)*	*strictCK*	*strictRK*	*strictCRK*
*base*	0.500 (0.535)	0.624	0.536	0.655

*base + PE*	0.530 (0.567)	0.654	0.566	0.684

*base + GP*	0.505 (0.539)	0.628	0.542	0.659

*base + PE + GP*	**0.536 **(0.571)	0.659	0.573	0.688

*PE: *predication equivalence, *GP: *gene/gene product correspondence, *CK: *conceptual knowledge, *RK: *relational knowledge, *CRK: *conceptual and relational knowledge

When we consider the indicator types that signal predications, modifier-head constructions (MOD_HEAD) appear more frequently as indicators than in the practice phase, as shown in Table 7. In addition, due to a clarification regarding how the indicator type for hypernymic relations should be annotated, we observed an increase in the frequency of the indicator type SPEC, which essentially indicates that the hypernymic relation between the concepts should be licensed by the UMLS Metathesaurus hierarchy in addition to being indicated by a textual clue.

**Table 7:** Top indicator types and their frequencies in the main annotation phase

Predicate Type	Average Count	A	B
PREP	462	467	457

VERB	243	257	229

MOD_HEAD	199	179	219

NOM	136	125	147

SPEC	101	96	106

The ontological predicate distribution in the main annotation phase is presented in Table 8. Comparing the distribution to that in the practice phase (as shown in Table 2), one noticeable trend is a relative increase in the number of PROCESS_OF, PART_OF, TREATS and AFFECTS predicates and a decrease in INHIBITS and INTERACTS_WITH predicates, possibly due to clarifications over definitions of the predicates after the practice phase. AFFECTS and INHIBITS predicates are not shown in Table 2 and Table 8, respectively, since they occur less frequently in the respective phases. However, we note that the increase in the number of AFFECTS predicates from the practice phase to the main annotation phase is 3.5%, whereas the decrease in that of INHIBITS is 8%. It is also worth mentioning that the high frequency of INHIBITS in the practice phase seems artificial, since most of those annotations were the result of a single instance that involved complex coordination.

**Table 8:** Most frequent ontological predicates and interannotator agreement specific to these predicates

Predicate	Average Count	%	A	B	IAA
PROCESS_OF	236	17.9	226	246	0.755

LOCATION_OF	199	15.0	198	200	0.578

PART_OF	164.5	12.5	150	179	0.500

TREATS	118.5	9.0	124	113	0.591

AFFECTS	104	7.9	88	120	0.308

ISA	101	7.7	96	106	0.593

CAUSES	57.5	4.4	52	63	0.561

USES	50.5	3.8	60	41	0.495

INTERACTS_WITH	46.5	3.5	61	32	0.366

ADMINISTERED_TO	35.5	2.7	26	45	0.500

During this phase, we also computed interannotator agreement (using *strict *agreement with *base + PE + GP *criterion) at the ontological predicate level to assess whether some types are more difficult to annotate than others. Among the most frequent predicates, there is less agreement on predicates relating biomolecular entities or processes (INTERACTS_WITH and AFFECTS, for example). On the other hand, predicates concerning disorders, anatomical parts and population groups yield overall higher agreement. Further examining ontological predicates with highest and lowest interannotator agreement (Table 9), these findings are confirmed. Among ontological predicates annotated more than 10 times, the highest disagreement rates were associated with those involving biomolecular entities and processes (all but PRECEDES are molecular-level predicates). On the other hand, the highest agreement rate was for PROCESS_OF and PREVENTS, both of which are disease-related ontological predicates. Similar topical trends were observable at the ontological predication level, as well (Table 10). Among those ontological predications annotated more than 10 times between annotators, the highest disagreement rates were found in those concerning the biomolecular entities and processes, while disagreement was lowest in predications involving disorders and population groups. These results clearly establish bio-molecular relations and disorder/population relations as two extremes in the spectrum in terms of ease of annotation.

**Table 9:** Highest and lowest agreement rates by ontological predicates, annotated more than 10 times

Predicate	IAA	Predicate	IAA
PROCESS_OF	0.755	INHIBITS	0.400

PREVENTS	0.667	PRODUCES	0.367

ISA	0.593	INTERACTS_WITH	0.366

TREATS	0.591	PRECEDES	0.320

DIAGNOSES	0.585	ASSOCIATED_WITH	0.320

LOCATION_OF	0.578	AFFECTS	0.308

CAUSES	0.561	STIMULATES	0.238

PART_OF	0.500	DISRUPTS	0.214

**Table 10:** Highest (column 1-3) and lowest (column 4-6) agreement rates for ontological predications annotated more than 10 times (N > 10)

Ontological Predication	N	IAA	Ontological Predication	N	IAA
*inpo-PROCESS_OF-humn *	15	0.947	*aapp-AFFECTS-celf *	13	0.375

*cell-PART_OF-mamm *	16	0.909	*aapp-INTERACTS_WITH-aapp *	16	0.222

*sosy-PROCESS_OF-humn *	28	0.866	*aapp-STIMULATES-aapp *	10	0

*dsyn-PROCESS_OF-humn *	141	0.859	*gngm-PART_OF-neop *	12	0

*neop-PROCESS_OF-humn *	54	0.857	*rcpt-PART_OF-neop *	11	0

*aapp: *Amino Acid, Peptide, or Protein; *celf: *Cell Function; *cell: *Cell; *dsyn: *Disease or Syndrome; *gngm: *Gene or Genome; *humn: *Human; *inpo: *Injury or Poisoning; *mamm: *Mammal; *neop: *Neoplastic Process; *rcpt: Receptor; sosy: *Sign or Symptom

Annotators were allowed to provide comments for their annotations, and we examined the comments corresponding to the predications on which there was disagreement. The most frequent comments in disagreement cases are given below. The number of disagreement instances and the corresponding interannotator agreement scores are given in parentheses. The interannotator agreement score for a specific type of comment was calculated by considering all the predications marked with that comment, regardless of whether there was disagreement or not.

a.** Unsatisfactory UMLS concept extracted by MetaMap: **The UMLS concept identified by MetaMap is replaced with a more appropriate concept from the UMLS 2006AA release. (275 instances, 0.307 IAA)

b. **No indicator rule: **There is no indicator rule mapping from the textual mention to the ontological predicate in SemRep. (213 instances, 0.292 IAA)

c. **Relational domain knowledge unavailable: **The annotated semantic predication does not correspond to an ontological predication in the SemRep ontology. (168 instances, 0.281 IAA)

d. **Newer UMLS release concept: **A non-2006AA UMLS concept is preferred. (163 instances, 0.278 IAA)

e. **Complete MetaMap miss: **A 2006AA UMLS concept that MetaMap fails to identify is used. (86 instances, 0.411 IAA)

### Adjudication Phase

In the adjudication phase, annotator C (adjudicator) examined the annotations provided by A and B, resolving differences and determining the gold standard. As well as selecting one annotation from one set over another from the other set, the adjudicator could also override annotations from both sets (generating new predications) or keep annotations from both sets (complementary predications). The final semantic predication count is 1371, an average of 2.74 predication per sentence. The maximum number of predications per sentence is 23. The frequency distributions by ontological predicate types and indicator types after reconciliation are presented in Table 11 and Table 12, respectively.

**Table 11:** Top ontological predicates and their annotation frequency in the gold standard

Ontological Predicate	Count
PROCESS_OF	239

LOCATION_OF	216

PART_OF	179

TREATS	126

ISA	111

AFFECTS	99

CAUSES	62

INTERACTS_WITH	51

USES	41

ADMINISTERED_TO	34

**Table 12:** Top indicator types and their frequencies in the gold standard

Indicator Type	Count
PREP	465

VERB	249

MOD_HEAD	217

NOM	141

SPEC	111

While interannotator agreement at this stage is not meaningful since the annotation sets were available to the adjudicator, we measured it to assess whether the adjudicator clearly prefers one set of annotation to the other. The adjudicator agreed with annotator B at a much higher rate (0.835 vs. 0.658). This seems likely due to the fact that the adjudicator took B annotations as the basis and only added an A annotation when B annotation was deemed incorrect.

## Discussion

We conducted a relatively open-ended, multi-phase annotation study, in which we aimed to assess the feasibility of iteratively constructing a reasonable gold standard reference based on UMLS domain knowledge. The results presented in the previous section show that this is a feasible undertaking. On the other hand, they confirm that conceptual annotation is extremely challenging; the main difficulty is mapping textual mentions to ontological terms (entities, processes, functions), a time-consuming and labor-intensive task. This is evidenced by the fact that interannotator agreement increases to acceptable levels (0.659) when the comparison is limited to conceptual knowledge available to the annotators. We discuss some of the challenges in more depth below.

### Mapping text to ontological concepts

The core notion in mapping textual mentions to ontological concepts is *semantic equivalence*, which entails that the ontological concept must express the meaning of the textual mention adequately, and should not correspond to a more general or more specific meaning than the textual mention. However, this equivalence is often very difficult to establish. In our study, the difficulty of establishing semantic equivalence is further compounded by the nature and size of the primary reference terminology we used for conceptual information, the UMLS Metathesaurus. In addition, we defined the task in an open-ended manner and allowed the annotators to use newer UMLS releases when the primary knowledge source (2006AA UMLS) is inadequate, which increased the complexity of the task and lowered the interannotator agreement.

To ease the burden for the annotator, we provided UMLS concepts identified by MetaMap as reference, corresponding to the domain knowledge available to SemRep. While this was useful to a large extent, it did not prevent disagreements regarding what constitutes an adequately expressive, semantically equivalent mapping. In some cases, the concepts identified by MetaMap may even be misleading, as we found out, since one annotator often considered a given concept adequate for the textual mention in question, while the other was dissatisfied with the mapping and identified a clearly better UMLS Metathesaurus concept. For example, MetaMap failed to completely map the noun phrase "the D3 receptor" to the existing UMLS concept "C0082341: dopamine D3 receptor" (Receptor) and identified the concept "C0597357: receptor" (Receptor) instead. One annotator considered this more general concept adequate, while the other identified the former as the more adequate mapping. A similar situation occurred with "minimally invasive surgery," where MetaMap identified "C0543467: Operative Surgical Procedures" (Therapeutic or Preventive Procedure), while, in fact, there exists a more suitable concept "C0282624: Surgical Procedures, Minimally Invasive" (Therapeutic or Preventive Procedure) for the mention. There were also cases where MetaMap failed to identify a concept corresponding to a textual mention, and it was difficult for the annotator to see that the textual mention may in fact have a UMLS counterpart. For instance, only one annotator found and used the concept "C1254042: Anatomical maturation (Physiologic Function)" to correspond to the head of the more specific noun phrase "lung maturation." Despite these difficulties, however, evidence from the annotators indicates that concepts identified by MetaMap were overall helpful to annotators and lightened the annotation load considerably. Furthermore, interannotator agreement increased significantly (approximately 12%) when only MetaMap-supplied concepts were used, supporting our basic intuition that selecting a concept from a predefined list is less demanding than searching for a semantically equivalent concept from a sizable terminology and assessing its adequacy, an essentially subjective task.

In other gold standard annotation studies, such as the CRAFT corpus and the CLEF corpus, carefully selected, well-curated vocabularies or ontologies were used. For example, the CRAFT corpus made use of six OBO ontologies. However, finding the semantically equivalent concept for a textual mention is still found to be challenging. In our annotation, consistent with the SemRep methodology, we did not limit ourselves to specific UMLS vocabularies and aimed to be more general in our coverage. However, this generality has its drawbacks, as expected. While synonymous terms from different vocabularies are clustered together to form concepts in the UMLS Metathesaurus, there are still a significant number of distinct concepts that are very close in meaning. For example, for the noun phrase "mineral metabolism impairment," MetaMap identified two separate concepts: "C0678715: mineral metabolism" (Organism Function) and "C0011155: Deficiency" (Functional Concept). Neither annotator found these mappings adequate (it can be argued that the best mapping would be a combination of both); however, they found different concepts, similar in meaning, to correspond to the meaning of the mention. One identified "C0687148: Mineral deficiency" (Disease or Syndrome), while the other identified "C0154260: Disorder of mineral metabolism" (Disease or Syndrome). While these concepts are not exactly the same, the difference between their meanings is arguably very small. A similar situation holds between "C0403716: Calculus in renal pelvis" (Disease or Syndrome) and "C0022650: Kidney Calculi" (Pathologic Function) for the phrase "pelvic stones." (note that "Pathologic Function" as a semantic type for "Kidney Calculi" seems incorrect; however, we did not judge the correctness of UMLS semantic type assignments in this work.) The problem seems even more acute when it comes to gene and gene product concepts, as exemplified earlier. It seems necessary to take into account such overlap and similarity in meaning, both in computing interannotator agreement and in system evaluation. We attempted to address the semantic equivalence of concepts to some extent by devising an equivalence criterion involving gene and gene products; however, this criterion is limited to a single, clear pattern, and is unable to accommodate more complex cases of equivalence. One principled solution may be to allow annotators to annotate more than one concept for a given textual mention when a clear preference for a concept cannot be established and to give partial (or full) credit for any of the concepts. This would clearly increase annotation complexity, and it may be more feasible as a postprocessing step.

### Annotation by domain

Our results also show that difficulty of annotation varies by the biomedical subdomain. Ontological predications relating to biomolecular entities and processes are most challenging to annotate, while those on the topic of population characteristics of disease seem to be the most straightforward. We explain the difficulty of annotating biomolecular relations by observing the following:

a. Molecular biology text is hardest to read and interpret for a non-expert and none of the annotators are experts in this subdomain. Furthermore, as Friedman et al. [37] have shown, biomolecular domain text constitutes a *sublanguage*, with very specific characteristics, such as complex and nested relations as well as more prevalent syntactic ambiguity. One interesting, syntactically ambiguous case involved the fragment "... IL-1beta-induced ROS formation, NF-kappaB activation, and MCP-1 secretion ...", where one annotator took the modifier "IL-1beta-induced" as modifying "NF-kappaB activation" and "MCP-1 secretion" as well as "ROS formation", and annotated the predications given in (6), while the other annotator took the modifier to modify "ROS formation" only, and did not annotate the predications in (6).

(6) C0021753: Interleukin-1 beta (Amino Acid, Peptide, or Protein)-STIMULATES-C0128897: Monocyte Chemoattractant Protein-1 (Amino Acid, Peptide, or Protein)

C0021753: Interleukin-1 beta (Amino Acid, Peptide, or Protein)-STIMULATES-C0079904: NF-kappa B (Amino Acid, Peptide, or Protein)

b. The coverage of the UMLS Semantic Network with respect to molecular biology is perhaps the least extensive. In fact, we have extended the UMLS Semantic Network to create the SemRep ontology in prior work [21,38] specifically to redress this gap.

c. Based on evidence from the annotation process, it seems more challenging for MetaMap to map textual mentions of biomolecular entities to UMLS Metathesaurus than to map those of entities from other subdomains. For example, the semantic type most frequently associated with concepts identified by annotators and not by MetaMap was *Amino Acid, Peptide, or Protein*.

In contrast to genomic concepts and relations, disorder and population group concepts and their relations are well covered in the UMLS and MetaMap has less difficulty in mapping text to these concepts. In addition, text concerning disease characteristics is overall easier to interpret for a non-expert.

### Diachronic change in domain knowledge

Another complicating factor in conceptual annotation is that the ontologies and vocabularies the concepts are derived from may change over time. There are two alternatives to address this situation: (a) using a static snapshot of the knowledge source (b) re-annotating at each update of the knowledge source. Our methodology was similar to the first alternative: we adopted the 2006AA release of UMLS as the primary source for conceptual information, while also recognizing that newer releases of UMLS have wider coverage, especially with respect to new drug or gene/protein names, thus allowing the annotators to use these resources, if necessary. Since we enforced text-bounded annotations, we believe that it will be possible to update the gold standard semi-automatically at future updates of the knowledge sources, using MetaMap or a similar program.

## Conclusions

We have presented the construction of a semantic predication gold standard from biomedical literature text using the conceptual annotation paradigm. Manual conceptual annotation is considered extremely challenging, and our results confirm this perception, while also confirming that reasonable interannotator agreement could be achieved iteratively, consistent with the findings of Bada et al. [4]. While the domain knowledge we used (UMLS) reflects the application-specific aspect of our annotation, we believe that our analysis and discussion provide important insights for future efforts in this area.

The resulting gold standard constitutes the first resource, to our knowledge, in the biomedical domain that incorporates conceptual annotation of semantic relations in a wide variety of subdomains. Two sets of annotations and the adjudicated gold standard are made publicly available [39] for research purposes. A UMLS license is required. The corpus size is relatively small and may be insufficient for training information extraction systems. However, we believe it can serve as a benchmark to evaluate independently developed systems based on UMLS knowledge sources. Our goal is to use it for this particular purpose, as well as to guide future system development.

## Authors' contributions

HK conceived of the study, led the annotation effort, performed the analyses, and drafted the manuscript. HK, GR, and MF contributed to annotation, guideline development, and helped draft the manuscript. TCR directed the research and helped draft the manuscript. All authors read and approved the final manuscript.

## Appendix

### Ontological Predicate Definitions

Ontological predicate definitions and an example for each of the definitions are provided below. Note that each of the following can also be negated (NEG_<predicate>):

• **ADMINISTERED_TO: **Given to an entity, when no assertion is made that the substance or procedure is being given as treatment.

***Patients **with single brain lesion received an extra 3 Gy x 5 **radiotherapy **...*. 

C0034618: Radiation therapy (Therapeutic or Preventive Procedure)-ADMINISTERED_TO-C0030705: Patients (Human)

• **AFFECTS: **Produces a direct effect on. Implied here is the altering or influencing of an existing condition, state, situation, or entity. This includes *has a role in*, *alters, influences, predisposes, catalyzes, stimulates, regulates, depresses, impedes, enhances, contributes to, leads to*, and *modifies*.

***BAP31 **and its caspase cleavage product regulate cell surface expression of tetraspanins and integrin-mediated **cell survival***.

C1424489: BCAP31 gene (Gene or Genome)-AFFECTS-C0007620: Cell Survival (Cell Function)

• **ASSOCIATED_WITH: **Has a relationship to (gene-disease relation).

***EP2 **plays a critical role in **tumorigenesis **in mouse mammary gland ..*.

C1419062: PTGER2 gene (Gene or Genome)-ASSOCIATED_WITH-C1326912: Tumorigenesis (Neoplastic Process)

• **AUGMENTS: **Expands or stimulates a process.

***Nicotine **induces** conditioned place preferences **over a large range of doses in rats*.

C0028040: Nicotine (Organic Chemical)-AUGMENTS-C0815102: place preference learning (Mental Process)

• **CAUSES: **Brings about a condition or an effect. Implied here is that an agent, such as for example, a pharmacologic substance or an organism, has brought about the effect. This includes *induces, effects, evokes*, and *etiology*.

***Neurocysticercosis **(NCC) is one of the major causes of **neurological disease **..*.

C0338437:Neurocysticercosis (Disease or Syndrome)-CAUSES-C0027765: nervous system disorder (Disease or Syndrome)

• **COEXISTS_WITH: **Occurs together with, or jointly.

*Food intolerance-related **constipation **is characterized by **proctitis***.

C0009806: Constipation (Sign or Symptom)-COEXISTS_WITH-C0033246: Proctitis (Disease or Syndrome)

• **CONVERTS_TO: **Changes from one form to another (both substances).

*... **plasma nitrite** is readily oxidized to **nitrate** within plasma ..*.

C0028137: Nitrites (Chemical Viewed Structurally)-CONVERTS_TO-C0699857: Nitrate (Inorganic Chemical)

• **COMPLICATES: **Causes to become more severe or complex, or results in adverse effects.

***Infections **can trigger GBS and exacerbate **CIDP***.

C0021311: Infection (Disease or Syndrome)-COMPLICATES-C0393819: Polyradiculoneuropathy, Chronic Inflammatory Demyelinating (Disease or Syndrome)

• **DIAGNOSES: **Distinguishes or identifies the nature or characteristics of.

***Manometry **showed a higher **anal sphincter resting pressure **..*.

C0024751: Manometry (Laboratory Procedure)-DIAGNOSES-C0429217: Anal sphincter pressure (Finding)

• **DISRUPTS: **Alters or influences an already existing condition, state, or situation. Produces a negative effect on.

*Overexpression of **NF-kappaB **inhibits tumor cell **apoptosis***.

C0079904: NF-kappaB (Amino Acid, Peptide, or Protein)-DISRUPTS-C0162638: Apoptosis (Cell Function)

• **INHIBITS: **Decreases, limits, or blocks the action or function of (substance interaction).

*In recent studies, the **BDNF **expression was reduced by typical **neuroleptics***.

C0040615: Antipsychotic Agents (Pharmacologic Substance)-INHIBITS-C0107103: Brain-Derived Neurotrophic Factor (Biologically Active Substance)

• **INTERACTS_WITH: **Substance interaction.

*Here we show that **chymases**, which are chymotryptic peptidases secreted by mast cells, hydrolyze **HGF **..*.

C0055673: Chymase (Enzyme)-INTERACTS_WITH-C0062534: Hepatocyte Growth Factor (Amino Acid, Peptide, or Protein)

• **ISA: **The basic hierarchical link in the UMLS Semantic Network. If one item *isa *another item then the first item is more specific in meaning than the second item.

*The sympathetic **neurotransmitter norepinephrine **has been found ..*.

C0028351: Norepinephrine (Neuroreactive Substance or Biogenic Amine)-ISA-C0027908: Neurotransmitters (Neuroreactive Substance or Biogenic Amine)

• **LOCATION_OF: **The position, site, or region of an entity or the site of a process.

*We report a case of primary cardiac **epithelioid hemangioendothelioma **arising from the **right atrium **of a 2-month-old infant*.

C1269890: Entire right atrium (Body Part, Organ, or Organ Component)-LOCATION_OF-C0206732: Hemangioendothelioma, Epithelioid (Neoplastic Process)

• **MANIFESTATION_OF: **That part of a phenomenon which is directly observable or concretely or visibly expressed, or which gives evidence to the underlying process. This includes *expression of, display of*, and *exhibition of*.

*Recurrence of **glomerulopathy **underlying** ESRD **was frequent for IgAN, FSG..*.

C1261469: End stage renal failure (Disease or Syndrome)-MANIFESTATION_OF-C1398810: glomerulopathy (Disease or Syndrome)

• **METHOD_OF: **The manner and sequence of events in performing an act or procedure.

*... because of the use of **SSCP **as a **screening method **and sequencing only a part of TSHR exon 10*.

C0243031: Single-Stranded Conformational Polymorphism (Laboratory or Test Result)-METHOD_OF-C0220908:Screening procedure (Health Care Activity)

• **OCCURS_IN: **Has incidence in a group or population.

***Older populations **are more prone to **bone loss **with weight loss ..*.

C0599877: loss; bone (Pathologic Function)-OCCURS_IN-C1518563: Older Population (Human)

• **PART_OF: **Composes, with one or more other physical units, some larger whole. This includes *component of, division of, portion of, fragment of, section of*, and *layer of*.

*The probasal bodies and **microtubules **within the **blepharoplast cavities**..*.

C0026046: Microtubules (Cell Component)-PART_OF-C0230744: Basal body of cilium or flagellum, not bacterial (Cell Component)

• **PRECEDES: **Occurs earlier in time. This includes *antedates, comes before, is in advance of, predates*, and *is prior to*.

*... the risk of tissue plasminogen activator-induced **hemorrhagic transformation **following **ischemic stroke **in mice ..*.

C0948008: Ischemic stroke (Disease or Syndrome)-PRECEDES-C1096400: Haemorrhagic transformation stroke (Disease or Syndrome)

• **PREDISPOSES: **To be a risk to a disorder, pathology, or condition.

... high **ghrelin **levels contribute to **obesity **in Prader-Willi syndrome (PWS) ...

C0911014: ghrelin (Amino Acid, Peptide, or Protein)-PREDISPOSES-C0028754: Obesity (Disease or Syndrome)

• **PREVENTS: **Stops, hinders or eliminates an action or condition.

***Ipsapirone **and ketanserin protects against circulatory shock, **intracranial hypertension**, and cerebral ischemia during heatstroke*.

C0123905: ipsapirone (Pharmacologic Substance)-PREVENTS-C0151740: Intracranial Hypertension (Disease or Syndrome)

• **PROCESS_OF: **Disorder occurs in (higher) organism.

*... no information is available in **CAD patients **with normal glomerular filtration rate (GFR)*.

C0010054: Coronary Arteriosclerosis (Disease or Syndrome)-PROCESS_OF-C0030705: Patients (Human)

• **PRODUCES: **Brings forth, generates or creates. This includes *yields, secretes, emits, biosynthesizes, generates, releases, discharges*, and *creates*.

*Human **EPCs **express functional **PAR-1**..*.

C0038250: Stem cells (Cell)-PRODUCES-C0668084:Receptor, PAR-1 (Amino Acid, Peptide, or Protein)

• **STIMULATES: **Increases or facilitates the action or function of (substance interaction).

***Candesartan **therapy significantly reduced inflammation and increased **adiponectin **levels ..*.

C0717550: candesartan (Pharmacologic Substance)-STIMULATES-C0389071: Adiponectin (Amino Acid, Peptide, or Protein)

• **TREATS: **Applies a remedy with the object of effecting a cure or managing a condition.

*This study initially surveyed 163 patients with clinical stage Ib or IIa **cervical adenocarcinoma **treated with **radical hysterectomy **and pelvic lymphadenectomy*.

C0677962: Radical hysterectomy (Therapeutic or Preventive Procedure)-TREATS-C0279672: Cervical Adenocarcinoma (Neoplastic Process)

• **USES: **Employs in the carrying out of some activity. This includes *applies*, *utilizes, employs*, and *avails*.

*Pre-emptive **therapy **with oral **valganciclovir **for CMV infections..*.

C0087111: Therapeutic procedure (Therapeutic or Preventive Procedure)-USES-C0909381: valganciclovir (Pharmacologic Substance)

• **COMPARED_WITH: **Comparative predicate.

*Compared with **placebo, candesartan **therapy significantly lowered plasma hsCRP levels..*.

C0032042: Placebos (Medical Device)-COMPARED_WITH-C0717550: candesartan (Pharmacologic Substance)

• **HIGHER_THAN: **Comparative predicate.

***Losartan **was more effective than **atenolol **in reducing cardiovascular morbidity..*.

C0126174: Losartan (Organic Chemical)-HIGHER_THAN-C0004147: Atenolol (Organic Chemical)

• **LOWER_THAN: **Comparative predicate.

***Amoxicillin-clavulanate **was not as effective as** ciprofloxacin **for treating uncomplicated bladder infection in women*.

C0054066: Amoxicillin-Potassium Clavulanate Combination (Antibiotics)-LOWER_THAN-C0008809: Ciprofloxacin (Pharmacologic Substance)

• **SAME_AS: **Comparative predicate.

***Candesartan **is as effective as** lisinopril **once daily in reducing blood pressure*.

C0717550: candesartan (Organic Chemical)-SAME_AS-C0065374: Lisinopril (Amino Acid, Peptide, or Protein)

### Predicate Types Abbreviations and Definitions

• **VERB: **verbal predicates

• **NOM: **nominalizations or other argument-taking nouns

• **ADJ: **adjectival predicates

• **PREP: **prepositional predicates

• **AUX: **auxiliary verb predicates

• **PART: **past participial predicates

• **MOD/HEAD: **the predication is the result of a noun phrase construction

• **SPEC: **the predication is hypernymic and must be licensed by the UMLS Metathesaurus hierarchy, that is, the subject must be a descendant of the object in the UMLS Metathesaurus hierarchy

• **INFER: **the predication is the result of inference based on two other existing predications

• **COMPLEX: **the indicator is a multi-word expression (e.g., "decrease the risk"), but not a multi-word lexical item (e.g., "risk factor").

### Sample Annotation

The following information (sentence and entities extracted by MetaMap and augmented by Entrez Gene lookup) is provided to the annotators:

*16397290.ab.8 High-dose aspirin at a therapeutic dose does not affect CYP2E1 activity in humans*.

C0444956|High dose|qnco|||High-dose|901|1|9

C0004057|Aspirin|orch,phsu|||aspirin|901|11|17

C0302350|Therapeutic|ftcn|||therapeutic|888|24|34

C1446466|Dose|qnco|||dose|888|36|39

C1518422|Not|ftcn|||not|1000|46|48

C0919426|CYP2E1 gene|gngm|1571|CYP2E1|CYP2E1|888| 57|62

C0439167|% activity|qnco|||activity|888|64|71

C0020114|Human|humn|||humans|1000|76|81

In the sentence line above, 16397290.ab.8 is a sentence identifier (8^th ^sentence of the abstract of the MEDLINE citation with PMID 16397290). In the subsequent entity lines, the fields correspond to the following (In square brackets are the corresponding elements of the underlined entity line above):

1. Concept identifier (CUI) *[C0919426]*

2. UMLS Metathesaurus preferred name *[CYP2E1 gene]*

3. UMLS semantic type abbreviation(s) *[gngm]*

4. Entrez Gene ID (if any) *[1571]*

5. Entrez Gene name (if any) *[CYP2E1]*

6. Textual mention that maps to the entity *[CYP2E1]*

7. MetaMap score for the entity *[888]*

8. First character position (in the sentence) of text denoting entity *[57]*

9. Last character position (in the sentence) of text denoting entity *[62]*

From these provided annotations, the following two semantic predication annotations are expected:

C0004057|Aspirin|orch,phsu|orch|||NEG_INTERACTS_WITH|C0919426|CYP2E1 gene|gngm,aapp|gngm|1571|CYP2E1|VERB|affect|aspirin|CYP2E1

C0020114|Human|grup,humn|humn|||LOCATION_OF|C0919426|CYP2E1 gene|gngm,aapp|aapp|1571|CYP2E1|PREP|in|humans|CYP2E1

Fields 1-3 and 8-10 correspond to fields 1-3 of the entity lines for subject and object, respectively. Fields 4 and 11 correspond to the individual semantic types that license the predication. Fields 5-6 and 12-13 correspond to fields 4-5 of the entity lines for subject and object, respectively. Field 7 corresponds to the ontological predicate (see the Section Ontological Predicate Definitions above). Field 14 is the ontological predicate type (see the Section Predicate Types above). Field 15 is the textual mention corresponding to the ontological predicate, field 16 corresponds to the subject argument and field 17 to the object argument.

## References

[B1] Björne J Ginter F Pyysalo S Tsujii J Salakoski T Scaling up Biomedical Event Extraction to the Entire PubMed Proceedings of the Workshop on Biomedical Natural Language Processing (BioNLP'10) 2010 28 36

[B2] Hristovski D Friedman C Rindflesch TC Peterlin B Exploiting semantic relations for literature-based discovery AMIA Annual Symposium Proceedings 2006 349 353 PMC1839258 17238361

[B3] Cohen T Whitfield GK Schvaneveldt RW Mukund K Rindflesch TC EpiphaNet: An Interactive Tool to Support Biomedical Discoveries Journal of Biomedical Discovery and Collaboration 2010 5 21 49 20859853 PMC2990276

[B4] Bada M Eckert M Palmer M Hunter L An Overview of the CRAFT Concept Annotation Guidelines Proceedings of the Fourth Linguistic Annotation Workshop 2010 207 211

[B5] Kim JD Ohta T Tateisi Y Tsujii J GENIA corpus - a semantically annotated corpus for bio-textmining Bioinformatics 2003 19 Suppl 1 180 182 10.1093/bioinformatics/btg1023 12855455

[B6] Pestian JP Brew C Matykiewicz P Hovermale D Johnson N Cohen KB Duch W A shared task involving multi-label classification of clinical free text Biological, translational, and clinical language processing 2007 97 104

[B7] Jimeno A Jimenez-Ruiz E Lee V Gaudan S Berlanga R Rebholz-Schuhmann D Assessment of disease named entity recognition on a corpus of annotated sentences BMC Bioinformatics 2008 9 Suppl 3 S3 10.1186/1471-2105-9-S3-S3 18426548 PMC2352871

[B8] Bunescu R Ge R Kate RJ Marcotte EM Mooney RJ Ramani AK Wong YW Comparative Experiments on Learning Information Extractors for Proteins and their Interactions Artificial Intelligence in Medicine (special issue on Summarization and Information Extraction from Medical Documents) 2005 33 2 139 155 10.1016/j.artmed.2004.07.016 15811782

[B9] Nédellec C Learning language in logic: genic interaction extraction challenge Proceedings of the ICML 2005 workshop: Learning Language in Logic (LLL05) 2005

[B10] Pyysalo S Ginter F Heimonen J Björne J Boberg J Järvinen J Salakoski T BioInfer: a corpus for information extraction in the biomedical domain BMC Bioinformatics 2007 8 50 10.1186/1471-2105-8-50 17291334 PMC1808065

[B11] Rosario B Hearst MA Classifying semantic relations in bioscience texts Proceedings of the 42nd Annual Meeting on Association for Computational Linguistics 2004 430 437

[B12] Roberts A Gaizauskas R Hepple M Extracting Clinical Relationships from Patient Narratives Proceedings of the Workshop on Current Trends in Biomedical Natural Language Processing 2008 10 18

[B13] Kim JD Ohta T Tsujii J Corpus annotation for mining biomedical events from literature BMC Bioinformatics 2008 9 10 10.1186/1471-2105-9-10 18182099 PMC2267702

[B14] Thompson P Iqbal SA McNaught J Ananiadou S Construction of an annotated corpus to support biomedical information extraction BMC Bioinformatics 2009 10 349 10.1186/1471-2105-10-349 19852798 PMC2774701

[B15] Rebholz-Schuhmann D Jimeno-Yepes A van Mulligen EM Kang N Kors J Milward D Corbett P Buyko E Beisswanger E Hahn U CALBC Silver Standard Corpus Journal of Bioinformatics and Computational Biology 2010 8 163 179 10.1142/S0219720010004562 20183881

[B16] Gene Ontology http://www.geneontology.org/

[B17] Kim JD Ohta T Pyysalo S Kano Y Tsujii J Overview of BioNLP'09 Shared Task on Event Extraction Proceedings of Natural Language Processing in Biomedicine (BioNLP) Workshop 2009 1 9

[B18] Kim JD Pyysalo S Ohta T Bossy R Tsujii J Overview of BioNLP Shared Task 2011 Proceedings of the BioNLP 2011 Workshop Companion Volume for Shared Task 2011 1 6

[B19] Rindflesch TC Fiszman M The interaction of domain knowledge and linguistic structure in natural language processing: interpreting hypernymic propositions in biomedical text Journal of Biomedical Informatics 2003 36 6 462 477 10.1016/j.jbi.2003.11.003 14759819

[B20] Bodenreider O The Unified Medical Language System (UMLS): integrating biomedical terminology Nucleic Acids Research 2004 32 Database 267 270 10.1093/nar/gkh061 PMC308795 14681409

[B21] Ahlers CB Fiszman M Demner-Fushman D Lang FM Rindflesch TC Extracting semantic predications from Medline citations for pharmacogenomics Pacific Symposium on Biocomputing 2007 209 220 17990493

[B22] McCray AT Srinivasan S Browne AC Lexical methods for managing variation in biomedical terminologies Proceedings of 18th Annual Symposium on Computer Applications in Medical Care 1994 235 239 PMC2247735 7949926

[B23] Smith LH Rindflesch TC Wilbur WJ MedPost: a part-of-speech tagger for biomedical text Bioinformatics 2004 20 14 2320 2321 10.1093/bioinformatics/bth227 15073016

[B24] Aronson AR Lang FM An overview of MetaMap: historical perspective and recent advances Journal of the American Medical Informatics Association 2010 17 3 229 236 20442139 10.1136/jamia.2009.002733 PMC2995713

[B25] Maglott D Ostell J Pruitt KD Tatusova T Entrez Gene: gene-centered information at NCBI Nucleic Acids Research 2005 33 Suppl 1 D54 D58 15608257 10.1093/nar/gki031 PMC539985

[B26] Tanabe L Wilbur WJ Tagging gene and protein names in biomedical text Bioinformatics 2002 18 8 1124 1132 10.1093/bioinformatics/18.8.1124 12176836

[B27] Kilicoglu H Fiszman M Rodriguez A Shin D Ripple A Rindflesch TC Semantic MEDLINE: A Web Application to Manage the Results of PubMed Searches Proceedings of the Third International Symposium on Semantic Mining in Biomedicine (SMBM 2008) 2008 69 76

[B28] Fiszman M Demner-Fushman D Kilicoglu H Rindflesch TC Automatic summarization of MEDLINE citations for evidence-based medical treatment: A topic-oriented evaluation Journal of Biomedical Informatics 2009 42 5 801 813 10.1016/j.jbi.2008.10.002 19022398 PMC2776079

[B29] Névéol A Lu Z Automatic integration of drug indications from multiple health resources ACM International Health Informatics Symposium (IHI) 2010 666 673

[B30] Open Biomedical Ontologies http://www.obofoundry.org/

[B31] Wattarujeekrit T Shah PK Collier N PASBio: predicate-argument structures for event extraction in molecular biology BMC Bioinformatics 2004 5 155 10.1186/1471-2105-5-155 15494078 PMC535924

[B32] Bizer C Heath T Berners-Lee T Linked Data - The Story So Far International Journal on Semantic Web and Information Systems 2009 5 3 1 22

[B33] Cohen J A Coefficient of agreement for nominal scales Educational and Psychological Measurement 1960 20 37 46 10.1177/001316446002000104

[B34] Hripcsak G Rothschild AS Agreement, the f-measure, and reliability in information retrieval Journal of American Medical Informatics Association 2005 12 3 296 298 10.1197/jamia.M1733 PMC1090460 15684123

[B35] UMLS Terminology Services http://uts.nlm.nih.gov/

[B36] Entrez Gene http://ncbi.nlm.nih.gov/gene

[B37] Friedman C Kra P Rzhetsky A Two biomedical sublanguages: a description based on the theories of Zellig Harris Journal of Biomedical Informatics 2002 35 222 235 10.1016/S1532-0464(03)00012-1 12755517

[B38] Rindflesch TC Libbus B Hristovski D Aronson AR Kilicoglu H Semantic relations asserting the etiology of genetic diseases AMIA Annual Symposium Proceedings 2003 554 558 PMC1480275 14728234

[B39] SemRep Gold Standard Annotation http://skr.nlm.nih.gov/SemRepGold

